# Having Hard Conversations: Leveraging Primary Care Social Workers to Improve Take-Up of VA Home Health Aide Services

**DOI:** 10.1093/geroni/igaf122.1773

**Published:** 2025-12-31

**Authors:** Lauren Hall, Margaret Ding, Emily Franzosa

**Affiliations:** James J Peters VA Medical Center, New York, New York, United States; James J Peters VA Medical Center, New York, New York, United States; Bronx VA, Bronx, New York, United States

## Abstract

The Veterans Health Administration’s (VHA) home health aide benefit (HHA) serves 180,000 Veterans annually and is VHA’s fastest growing and most widely used home and community-based service. Like other payers, VHA purchases HHA care from community providers, which can make navigating the benefit a complex process for VHA staff and veterans. We conducted multi-perspective, semi-structured interviews at five geographically diverse VHA medical centers with key informants (n = 61) who connect veterans to HHA services, including primary care providers (geriatricians, social workers, nursing staff), HHA program managers, and home health agency administrators and aides to identify best practices and modifiable intervention points to improve take-up of VA HHA services. Interviews were recorded, transcribed, and analyzed using inductive thematic analysis. Findings across all participants emphasized the importance of primary care social workers in assisting veterans in making the most informed decisions about whether to use HHA. We identified 3 main themes that facilitated Veterans’ use of HHA: 1) social workers often are the primary care team members who know the veteran and their needs best; 2) social workers use rapport-building, goal alignment, and priority setting to persuade veterans to take up services, and 3) valuing veterans’ autonomy helps establish trust and support, encouraging veterans’ use of HHA. Leveraging the central role that social workers play in geriatric primary care teams, their relationships with older patients, and the recognition of veterans’ autonomy and preferences can support age-friendly care and improve older adults’ connection to the supports they need to live independently.

